# Traceback: leveraging archived biospecimens to identify mutation carriers

**DOI:** 10.18632/oncotarget.21742

**Published:** 2017-10-11

**Authors:** Goli Samimi, Mark E. Sherman

**Affiliations:** Goli Samimi: Division of Cancer Prevention, National Cancer Institute, Bethesda, MD, USA

**Keywords:** traceback, ovarian/tubal cancer, serous tubal intraepithelial carcinoma, BRCA1/2

In 2007, The National Comprehensive Cancer Network (NCCN) provided guidelines recommending *BRCA1/2* genetic testing for women diagnosed with ovarian, tubal or primary peritoneal cancers [1]. However, studies using data from 3 pooled cross-sectional National Health Interview surveys (2005, 2010, 2015) indicate that <20% of these women have undergone genetic testing, which equates to an estimated 400,000 ovarian cancer patients in the United States with undefined mutation status [2]. Furthermore, compliance rates based on specific diagnoses (ovarian *versus* tubal *versus* peritoneal cancer) are unclear. Low rates of genetic testing reflect missed opportunities in a complex sequence beginning with patient-physician discussions and referral, genetic counseling and risk assessment and culminating in genetic testing and post-test counseling.

Approaches are needed to increase the identification of women with *BRCA1/2* related gynecologic cancers so that these individuals can receive genetic counseling and testing, and access effective preventive interventions, if indicated. Importantly, identification of probands offers an important opportunity to educate entire families, thereby providing benefit to multiple individuals. In response to this opportunity, the National Cancer Institute conducted a workshop to discuss a framework for increasing identification of hereditary breast and ovarian cancer (HBOC) families termed “Traceback”. A key component of Traceback is the identification of archived ovarian, tubal and peritoneal cancer pathology specimens, which typically include uninvolved tissues that provide a source of germline DNA for *BRCA1* and *BRCA2* mutation testing [3]. A strength of this approach is that it may facilitate identification and counseling of individuals within mutation carrier families who live in medically underserved communities and are unaware of their increased risk of developing potentially lethal, yet preventable cancers.

Although the high fatality rate of high-grade ovarian/tubal cancers and patient mobility pose challenges for contacting women diagnosed in past years, relying on archived tissues may partly overcome this obstacle. As the ability to test for genetic risk markers expands, a successful Traceback approach could be applied to many different genetic mutations and tumor types.

The ethical and legal issues with respect to contacting next-of-kin, as well as other challenges associated with identifying probands through analysis of pathology specimens (*e.g*. availability of tissue blocks and limitations of testing archival biospecimens) have been described elsewhere [3]. Another major challenge with respect to this approach is that it relies heavily on accurate diagnosis and pathology reporting in order to find potential carriers. Paradoxically, dramatic revisions in our understanding of the pathogenesis of ovarian cancer offer new opportunities for prevention, while at the same time creating confusion about the clinical significance of recently described pathologic entities.

Historically, high-grade serous carcinoma, the most lethal type of ovarian carcinoma, was presumed to arise from the ovarian surface epithelium. Although efforts to identify an ovarian cancer precursor proved futile, this failure was ascribed to destruction of normal anatomy by large tumor masses. Introduction of risk-reducing surgery among *BRCA1* and *BRCA2* mutation carriers afforded pathologists an opportunity to examine fallopian tubes of high-risk women with intact microanatomy, which led to the discovery of small occult tubal carcinomas [4]. In the early 2000s, the Sectioning and Extensively Examining the Fimbriated End (SEE-Fim) pathology processing protocol was developed to enable comprehensive histopathologic study of fallopian tubes from risk-reducing bilateral salpingo-oophorectomy specimens, which led to increasing recognition of occult foci of high-grade cancer in the mucosa of the tubal fimbria, now termed “serous tubal intraepithelial carcinoma (STIC)” [5]. Thus, many tumors hitherto considered “ovarian” cancers were suggested to in fact represent primary tubal cancers, although the biology of tubal lesions remains ill-defined. This paradigm shift in our understanding has immense implications for improved risk reduction, early detection, diagnosis and staging, and treatment strategies.

Data indicate that diagnoses of fallopian tube cancers have increased dramatically in recent years [6], likely reflecting increased histologic examination of fallopian tube fimbriae removed during surgeries for cancer, risk-reduction or incidentally with hysterectomy, and increased recognition of STICs as early forms of tubal cancer [7]. As knowledge of STIC increased in the United States, performance of bilateral salpingectomy increased 77% (2000 to 2013) and hysterectomy with bilateral salpingectomy increased 4-fold (1998 to 2001) [8]. These clinical procedures could lead to increased detection of curable tubal cancers and STIC, as well as prevention of cancers by removal of fallopian tubes prior to tumor development. Although questions regarding the biology of STIC remain, offering genetic testing to patients with STIC may offer important benefits to patients and their relatives.

Pathologists may increasingly diagnose high grade serous carcinomas associated with STIC as tubal carcinomas, whereas prior to recognition of STIC, most of these tumors would likely have been diagnosed as ovarian cancers. Therefore, diagnoses of ovarian cancer may decline, whereas reports of tubal cancer will continue to increase. As progress is made towards understanding the pathogenesis of high-grade serous carcinoma and developing more effective treatments, we must not lose sight of the tremendous opportunity afforded by a growing list of highly promising prevention strategies. Specifically, pairing appropriate use of salpingectomy, either for prevention or opportunistically at the time of benign gynecologic procedures, with effective Traceback approaches may offer a long-sought opportunity to make quantum leaps in ovarian/tubal cancer prevention.
